# ICAM-1-binding *Plasmodium falciparum* erythrocyte membrane protein 1 variants elicits opsonic-phagocytosis IgG responses in Beninese children

**DOI:** 10.1038/s41598-022-16305-0

**Published:** 2022-07-29

**Authors:** Jennifer Suurbaar, Azizath Moussiliou, Rachida Tahar, Rebecca W. Olsen, Yvonne Adams, Nanna Dalgaard, Eric K. Baafour, Selorme Adukpo, Lars Hviid, Kwadwo A. Kusi, Jules Alao, Michael F. Ofori, Nicaise T. Ndam, Anja R. Jensen

**Affiliations:** 1 Department of Immunology, Noguchi Memorial Institute for Medical Research, University of Ghana, Legon, Ghana; 2 Department of Biochemistry, Cell and Molecular Biology, West African Centre for Cell Biology of Infectious Pathogens, University of Ghana, Legon, Ghana; 3 Department of Biochemistry and Molecular Medicine, School of Medicine and Health Sciences, University for Development Studies, Tamale, Ghana; 4 Centre for Medical Parasitology at Department of Immunology and Microbiology, Faculty of Health and Medical Sciences, University of Copenhagen, Copenhagen, Denmark; 5 Université de Paris Cité, MERIT, IRD, 75006 Paris, France; 6 Department of Pharmaceutics and Microbiology, School of Pharmacy, University of Ghana, Legon, Ghana; 7 Department of Infectious Diseases, Copenhagen University Hospital (Rigshospitalet), Copenhagen, Denmark; 8 Paediatric Department, Mother and Child University and Hospital Center (CHUMEL), Cotonou, Benin

**Keywords:** Infectious diseases, Malaria, Immunology, Adaptive immunity, Humoral immunity, Antibodies

## Abstract

Members of the highly polymorphic *Plasmodium falciparum* erythrocyte membrane protein 1 (PfEMP1) family expressed on the surface of infected erythrocytes (IEs) are important virulence factors, which mediate vascular adhesion of IEs via endothelial host receptors and are targets of naturally acquired immunity. The PfEMP1 family can be divided into clinically relevant subgroups, of which some bind intercellular adhesion molecule 1 (ICAM-1). While the acquisition of IgG specific for ICAM-1-binding DBLβ domains is known to differ between PfEMP1 groups, its ability to induce antibody-dependent cellular phagocytosis (ADCP) is unclear. We therefore measured plasma levels of DBLβ-specific IgG, the ability of such IgG to inhibit PfEMP1-binding to ICAM-1, and its ability to opsonize IEs for ADCP, using plasma from Beninese children with severe (SM) or uncomplicated malaria (UM). IgG specific for DBLβ from group A and B ICAM-1-binding PfEMP1 were dominated by IgG1 and IgG3, and were similar in SM and UM. However, levels of plasma IgG inhibiting ICAM-1-binding of group A DBLβ of PFD1235w was significantly higher in children with UM than SM, and acute UM plasma induced a higher ADCP response than acute SM plasma.

## Introduction

Malaria is one of the most important public health problems worldwide with an estimated global number of 241 million cases and 627 000 deaths (2020), the majority in children less than five years of age^1^. To help combat malaria, WHO in October 2021 recommended the widespread use of the RTS,S/AS01 vaccine in areas of moderate to high *P. falciparum* transmission^2^, and other vaccine candidates are in clinical trials including two (PAMVAC and PRIMVAC) based on *P. falciparum* erythrocyte membrane protein 1 (PfEMP1)^3^. Antibodies against variant surface antigens on the surface of infected erythrocytes (IEs) are known to protect individuals from severe and symptomatic malaria^4–6^, and members of the PfEMP1 family have been shown to be targets of naturally acquired antibodies^5,7–10^. The highly polymorphic PfEMP1 antigens are encoded by approximately 60 *var* genes per haploid parasite genome, mediate adhesion of IEs to vascular host receptors, and are associated with the pathogenesis of *P. falciparum* malaria^11^. Despite their extensive variation, the *var* genes can be divided into three major groups (A, B, and C) based on chromosome location, DNA sequence, and promotor region^12,13^. Chimeric group B/A genes exist, and together with group A *var* genes, are transcribed in parasites isolated from children with severe disease, and in cytoadhering IEs linked to the pathogenesis of cerebral malaria (CM). A subgroup of group A and B/A PfEMP1 proteins can bind both to the intercellular adhesion molecule 1 (ICAM-1) and endothelial protein receptor C (EPCR), and this dual-binding phenotype has been linked to CM^14,15^ but not all studies^16^. Infected erythrocytes co-localize with ICAM-1 expression in the brain blood vessels suggesting that ICAM-1 mediates IE sequestration in CM^17^. Dual-receptor binding IEs have been shown in vitro to cause clustering of ICAM-1, to be taken up by brain endothelial cells in an ICAM-1-dependent manner, that results in the breakdown of the blood–brain barrier and swelling of endothelial cells, and antibodies directed to the PfEMP1 on their surface prevent sequestration of IEs^18^. Sequestration of large numbers of IEs in the microvasculature of specific organs is central to the pathogenesis of severe *P. falciparum* malaria^19^. CIDRα domains associated with CM^20,21^ are the focus of some research groups, while we focus on DBLβ domains. In this study, we hypothesized that children with uncomplicated malaria (UM) have higher levels of functional anti-PfEMP1 (anti-DBLβ domain) antibodies than severe malaria (SM) cases and because of this, they would have antibodies against PfEMP1 variants associated with severe disease. To test this, we measured plasma levels of anti-PfEMP1 (DBLβ)-IgG in a cohort of Beninese children with SM or UM and used ICAM-1-binding inhibition and ADCP as measures of antibody effector function**.**

## Results

### IgG specific for DBLβ domains in ICAM-1-binding PfEMP1 variants

**Figure 1:**
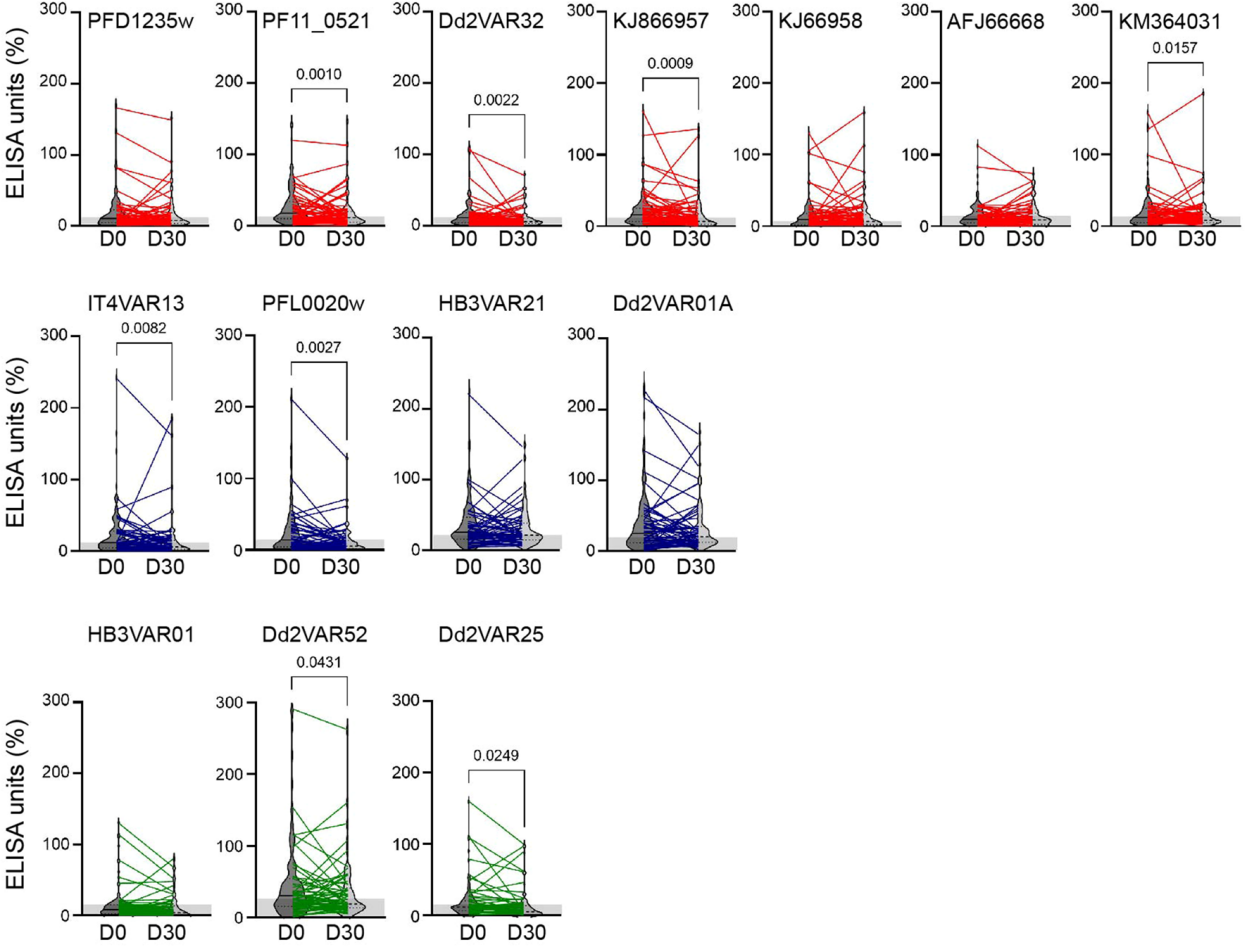
Plasma levels of IgG with specificity for *P. falciparum* DBLβ domains. Samples were obtained from 137 Beninese children at hospitalization (day 0) and at convalescence (day 30) following discharge from the hospital. Antibody levels (ELISA units; EU) in plasma from individual children against the ICAM-1-binding DBLβ of group A (top panel) PFD1235w, PF11_0521; Dd2VAR32, KJ866957, KJ866958, AFJ66668, KM364031, ICAM-1-binding group B (middle panel) IT4VAR13, PFL0020w, HB3VAR21, Dd2VAR01A, and non-ICAM-1-binding group A (bottom panel) DBLβ domains of HB3VAR01, Dd2VAR52, and Dd2VAR25. The violin plots show the median and interquartile range of all-day 0 and day 30 samples, lines show day 0 and day 30 values of paired samples i.e., same individual. Statistical significance comparing day 0 and day 30 was determined using Wilcoxon matched pairs signed rank test and significant *P-*values shown along the top of the panels. Thirty (30) non-exposed Danish individuals were included as negative controls (DK); the grey shaded area indicates sample reactivity below the cut-off (DK mean + 2SD).

**Figure 2:**
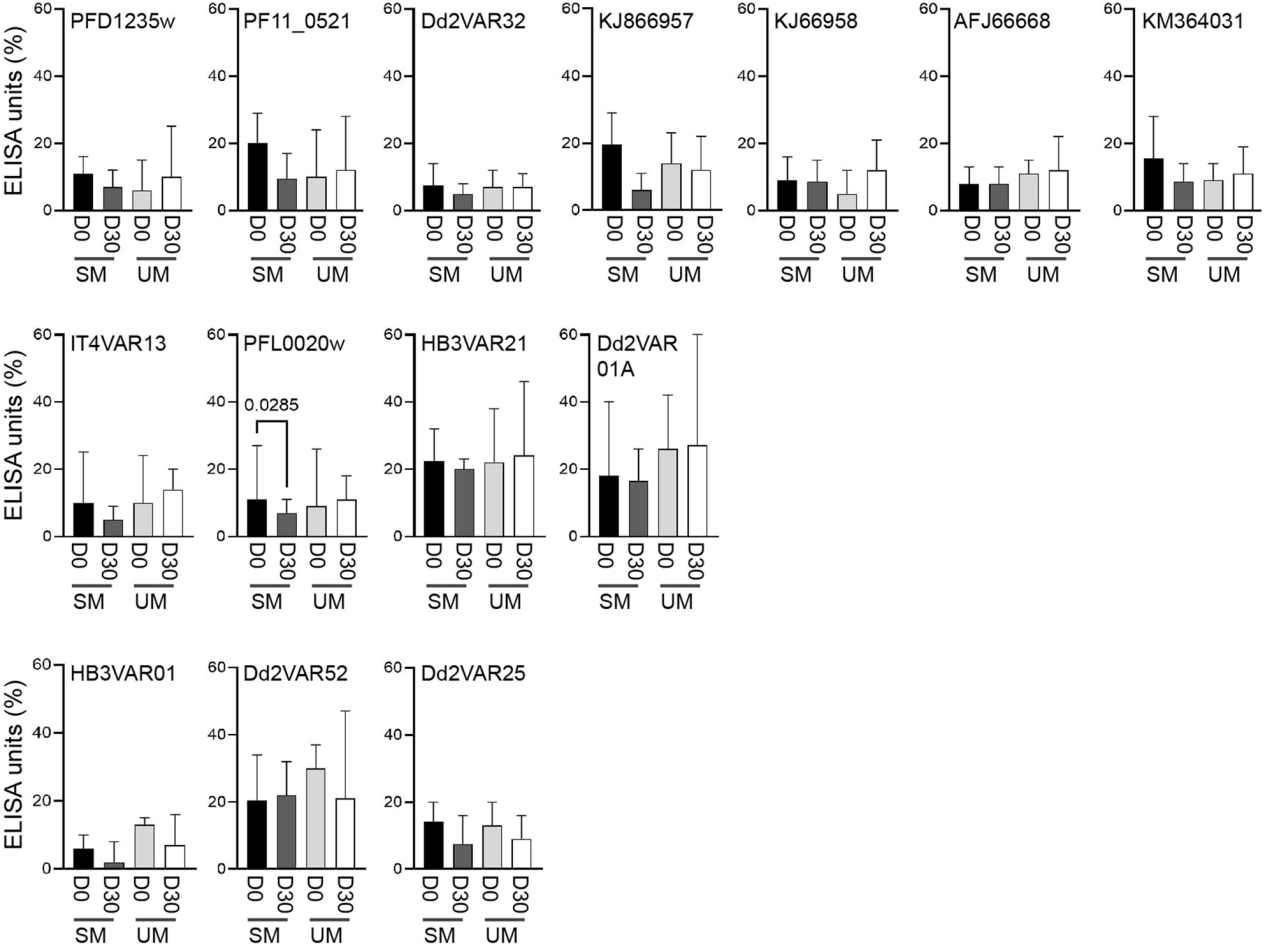
Plasma IgG levels against DBLβ in children in matched children´s samples and clinical categories. The anti-DBLβ antibody reactivity (ELISA units; EU) of the individual children shown in Fig. 1 were grouped according to clinical disease category, i.e., severe malaria (SM including CM and nCSM; *n* = 28), and uncomplicated malaria (UM, *n* = 27) at the day 0 (hospitalization) and day 30 (convalescent) samples. The reactivity was measured against the same DBLβ domains as in Fig. 1. Statistical significance comparing the different groups was determined using Wilcoxon matched pairs signed rank test, no significant differences found.

To investigate the anti-PfEMP1-IgG antibody reactivity against ICAM-1-binding DBLβ domains, 137 Beninese children (median age 36 months; IQR 22 to 48 months) were recruited at three different hospital centers and divided into three clinical categories: cerebral malaria (CM), non-cerebral severe malaria (nCSM), and uncomplicated malaria (UM) (Table 1). The mortality rate of CM cases was 26.5% versus 2% among children with nCSM or UM. The mean hemoglobin level of UM was significantly higher than that of children with CM or nCSM (P < 0.0001, Table 1). We assessed IgG levels against seven group A and four group B ICAM-1-binding DBLβ domains, and against three non-ICAM-1-binding group A DBLβ domains (Fig. 1). The anti-DBLβ-IgG levels among the children were significantly higher at hospitalization (day 0) than at convalescence (day 30) for four group A ICAM-1-binding DBLβ domains (PF11_0521, Dd2VAR32, KJ866957, KM364031), two group B ICAM-1-binding domains (IT4VAR13, PFL0020w) and two group A non-ICAM-1-binding PfEMP1 domains (Dd2VAR25, Dd2VAR52) (Fig. 1). We did not find any significant differences in reactivity between SM (CM and nCSM) and UM (Fig. 2).

**Table 1:** Characteristics of study participants^1^.

Characteristic	CM (n = 34)	nCSM (n = 51)	SM (n = 85)	UM (n = 52)	P-value
Age (months)	42.0 (30–49.5)	36 (18–48)	36 (21–48)	36 (24–48)	0.1118
Males/females (%)	50/50	58.8/41.2	56.3/43.7	63.5/36.5	0.1730
Hemoglobin	5.40 (3.75 – 7.23)	4.6 (3.6–6.2)	4.65 (3.6–6.3)	8.85 (7.5–10.35)	< 0.0001
Blantyre coma score	2 (2–2)	5 (3–5)	4 (3–5)	NA	
Parasitemia	48,000 (2010–257,250)	69,987 (12,800–346,265)	64,000 (4977–567,003)	55,156 (11,986–158,000)	0.5212
Mortality rate (%)	26.5	1.9		1.9	

*SM* all severe malaria cases, i.e., CM and nCSM; *CM* cerebral malaria, *nCSM* non-cerebral severe malaria, *UM* uncomplicated malaria. Hemoglobin (g/dL); Parasitemia (parasites/µl). Values are medians (25th; 75th percentile). Kruskal–Wallis’s test was used to compare the three groups (CM, nCSM and UM).

### Subclass IgG antibody responses

**Figure 3:**
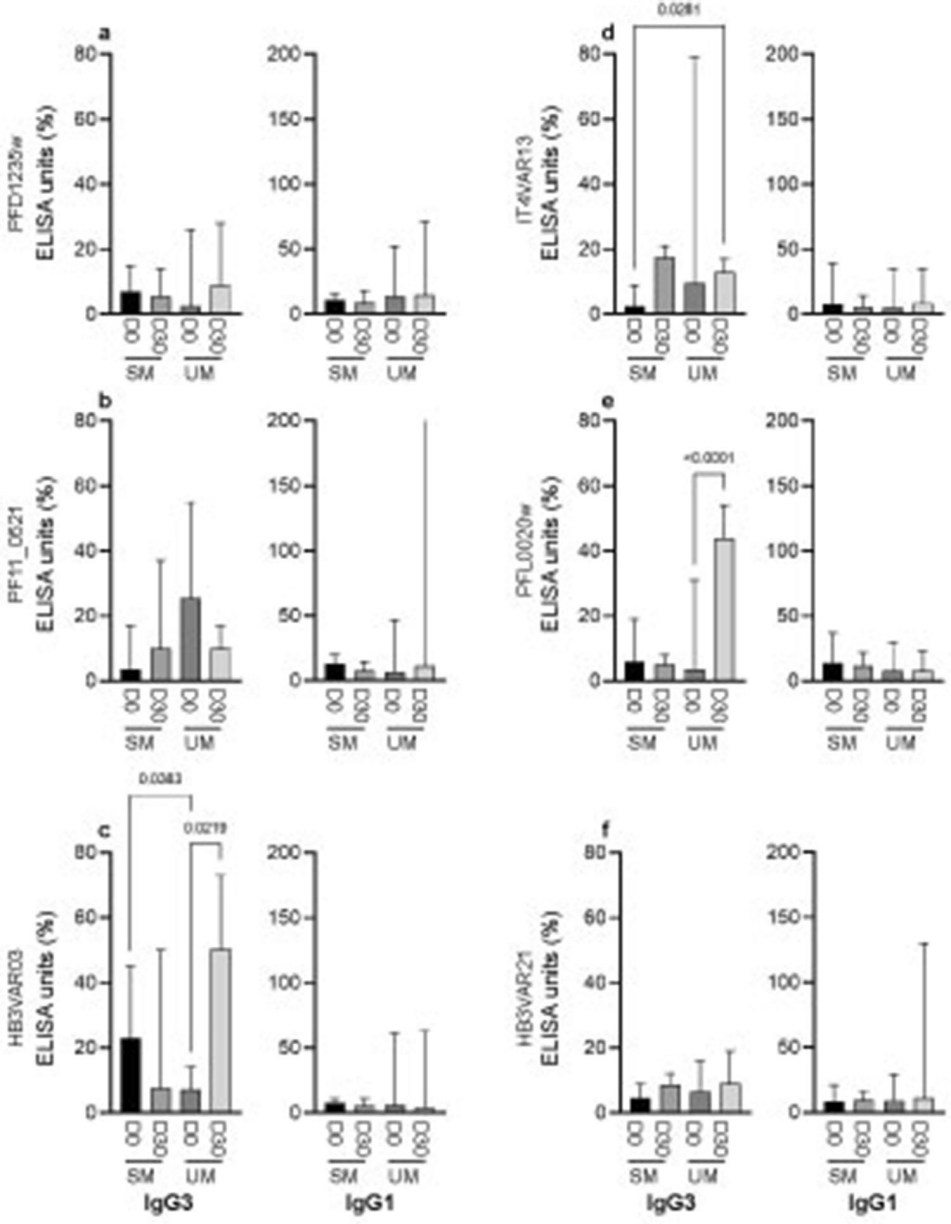
IgG subclass reactivity to group A and B ICAM-1-binding DBLβ domains. IgG1-4 antibody levels (ELISA units; EU) were measured in day 0 plasma samples from 84 of 137 Beninese children against DBLβ of group A (**a**) PFD1235w, (**b**) PF11_0521, (**c**) HB3VAR03, and group B (**d**) IT4VAR13, (**e**) PFL0020w, and (**f**) HB3VAR21. The anti-DBLβ antibody reactivity (ELISA units; EU) of the individual children were grouped according to clinical disease category, i.e., severe malaria (SM including CM and nCSM), and uncomplicated malaria (UM). The IgG2 and IgG4 median EU levels against any of the proteins were ≤ 9 EU (data not shown). Bars indicate the median and interquartile range. Statistical significance was determined using Wilcoxon matched pairs signed rank test and significant *P*-values shown along the top of the panel.

To determine whether the antibody subclass response against different ICAM-1-binding DBLβ domains differed, we measured both cytophilic (IgG1, IgG3) and non-cytophilic (IgG2, IgG4) IgG in plasma samples from 84 (day 0) and 45 (day 30) of the children. Cytophilic IgG completely dominated the anti-DBLβ (PFD1235w, PF11_0521, HB3VAR03, HB3VAR21, PFL0020w, IT4VAR13) antibody response in all children (Fig. 3), as IgG2 and IgG4 levels were low to undetectable (data not shown). The dominance of IgG1 and IgG3 suggests that antibody-mediated protection against SM relies not only on inhibition of IE sequestration (neutralization), but also involves ADCP and possibly activation of the classical complement cascade. Levels of HB3VAR03-specific IgG3 were significantly higher in children with SM (day 0, *P* = 0.04) compared to children with UM (Fig. 3c). A similar trend was seen for PFD1235w-specific IgG3, but this did not reach statistical significance (*P* = 0.09) (Fig. 3a). The IgG1 and IgG3 antibody response against PF11_0521 (group A), and group B DBLβ domains (HB3VAR21, PFL0020w, IT4VAR13) did not differ between the two clinical categories (Fig. 3b,d–f).

### Adhesion-inhibitory plasma IgG

**Figure 4:**
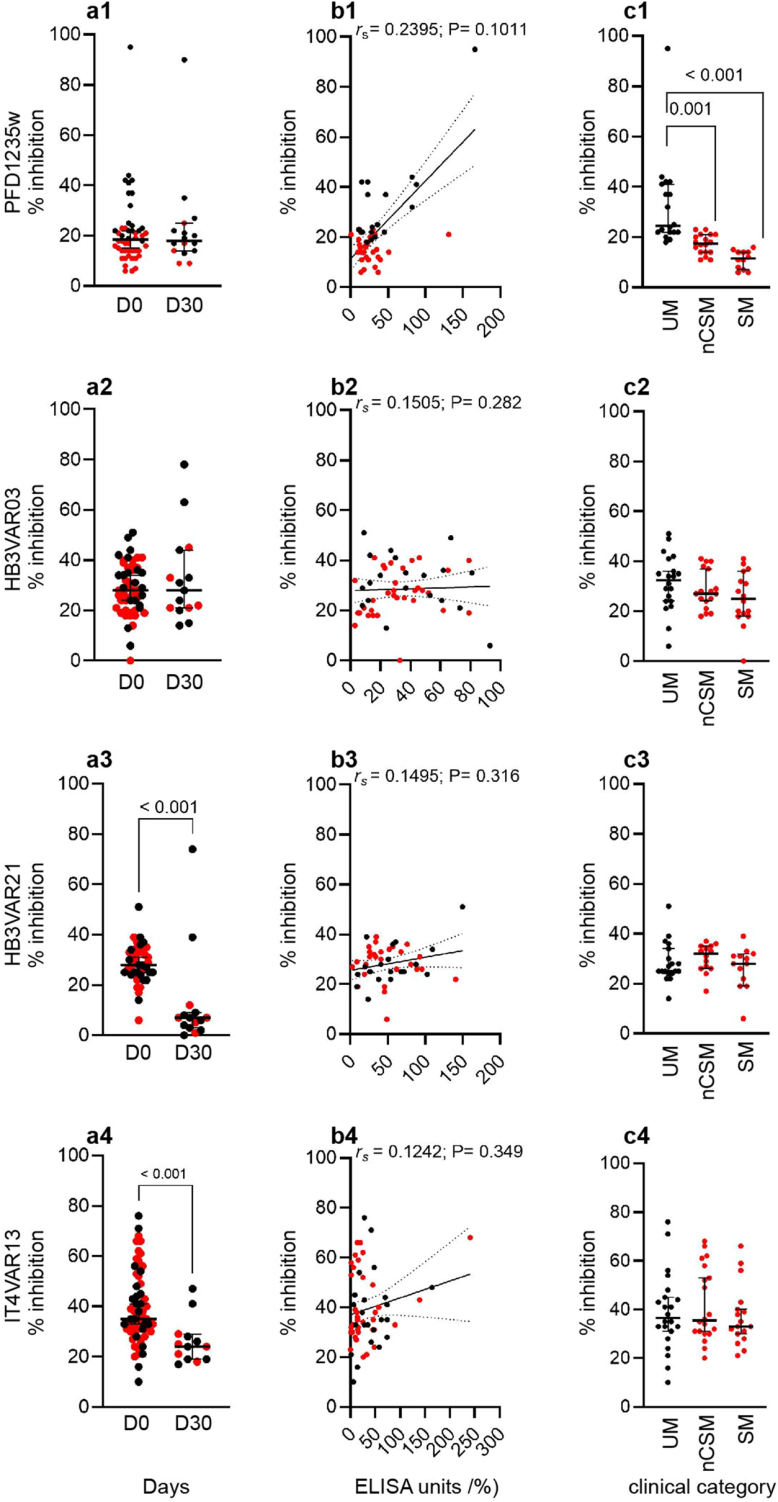
Plasma IgG inhibition of PfEMP1 DBLβ domain binding to ICAM-1. The percentage (%) binding inhibition of individual plasma samples from Beninese children collected at hospitalization (day 0) and at convalescence (day 30) and for different clinical categories (CM, nCSM, UM) at hospitalization. (**a**) The percentage (%) binding inhibition of individual plasma samples were calculated compared to ICAM-1-binding of DBLβ in the absence of plasma. The inhibition was measured for group A (**a1**) PFD1235w [(day 0, *n* = 50; day 30, *n* = 16), (CM, *n* = 13; nCSM, *n* = 19; UM, *n* = 19)] and (**a2**) A HB3VAR03 [(day 0, *n* = 53; day 30, *n* = 14), (CM, *n* = 16; nCSM, *n* = 17; UM, *n* = 20)], group B (**a3**) HB3VAR21 [(day 0, *n* = 50; day 30, *n* = 15), (CM, *n* = 13; nCSM, *n* = 14; UM, *n* = 23)] and (**a4**) IT4VAR13 [(day 0, *n* = 60; day 30, *n* = 13) (CM, *n* = 16; nCSM, *n* = 20; UM, *n* = 24)]. Statistical significance was determined using determined using Wilcoxon matched pairs signed rank test and significant *P*-values shown along the top of each panel. Black lines indicate the median and interquartile range. (**b**) The percentage inhibition of individual samples was correlated to the antibody reactivity (EU %) of each individual sample (day 0) using Spearman correlation test. Spearman´s correlation coefficient (r_s_) and *P-*value are shown in each panel, (**c**) The percentage ICAM-1-binding inhibition of plasma antibodies shown for each of the three clinical categories uncomplicated (UM), severe (SM), and cerebral malaria (CM) at day 0. Lines indicate the median and interquartile range. Red dots represent SM and/or CM samples.

The plasma samples (day 0, *n* = 49 to *n* = 60; day 30, *n* = 14 to *n* = 17) showed varying adhesion inhibitory capacity against DBLβ domains of PFD1235w, HB3VAR03, HB3VAR21, and IT4VAR13. At day 0, the percentage of plasma samples showing ≥ 30% inhibition of the ICAM-1-binding of the DBLβ domains was 16% (8/49; PFD1235w), 41% (22/54; HB3VAR03), 43% (20/47; HB3VAR21), and 78% (47/60; IT4VAR13) (Fig. 4a1–a4). The ability to inhibit binding of the PfEMP1 domains to ICAM-1 did not correlate significantly with the IgG antibody level against the specific DBLβ domain (Fig. 4b1–b4). From this analysis, we proceeded to stratify our study participants into the three clinical categories CM, nCSM, and UM to assess the potential inhibitory role of their plasma (Fig. 4c). Although the median anti-PFD1235w IgG level of the different disease categories did not differ significantly (Fig. 3a)**,** the median percentage plasma IgG inhibition of PFD1235w DBLβ binding to ICAM1 (Fig. 4c1) was significantly higher for UM than nCSM (*P* = 0.001) or CM (*P* < 0.001), while this was not the case for the DBLβ domain of HB3VAR03 (group A) or HB3VAR21 and IT4VAR13 (group B) (Fig. 4c2–c4).

### Antibody-dependent cellular phagocytosis

**Figure 5:**
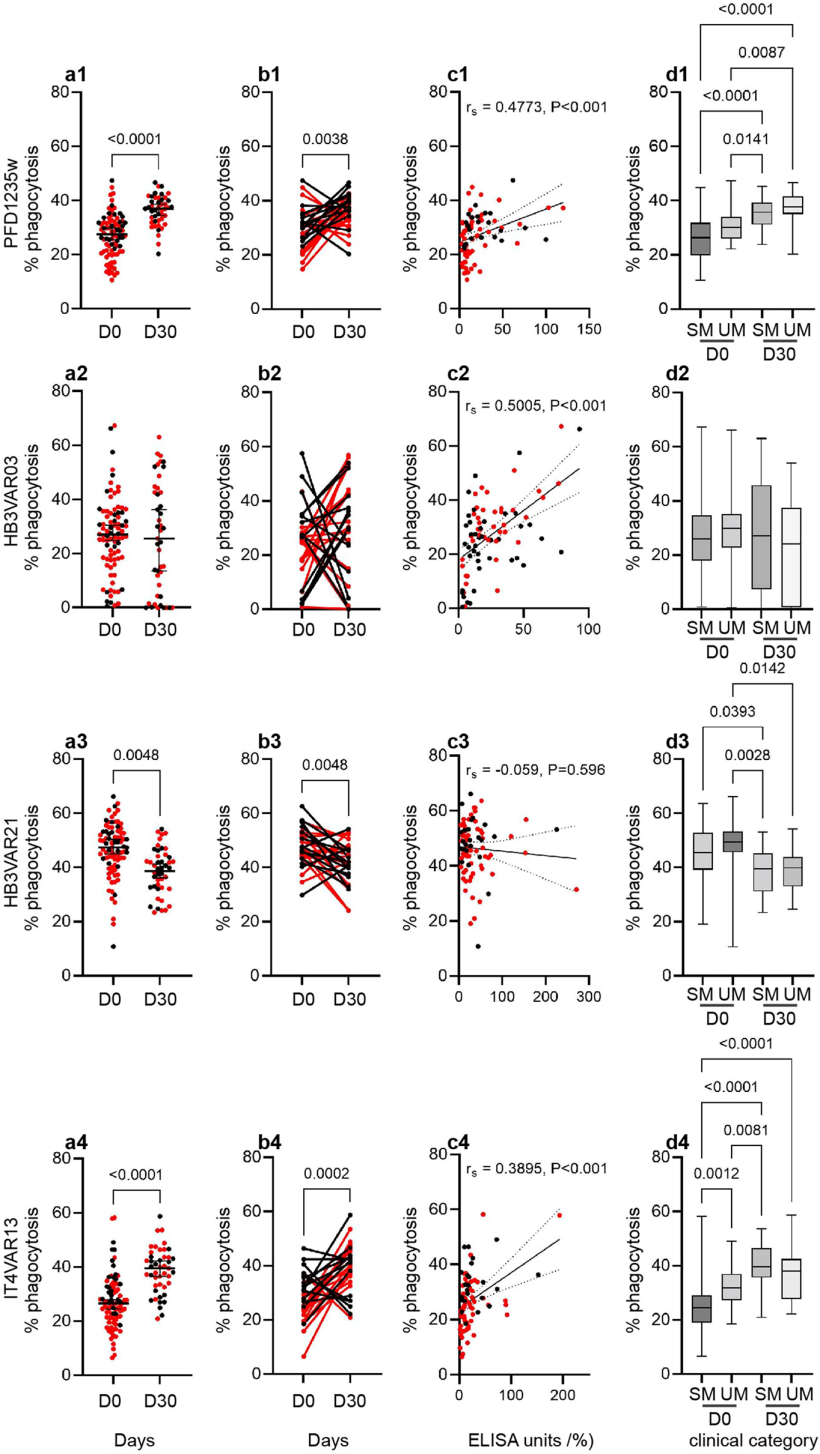
Plasma IgG-mediated phagocytosis of DBLβ coated-beads. (**a**) The percentage (%) phagocytosis by individual plasma samples from Beninese children collected at hospitalization (day 0, *n* = 84) and at follow-up (day 30, *n* = 45) were calculated as the percentage of THP-1 cells that internalized coupled beads. Beads were coupled with either (**a1**) PFD1235w, (**a2**) HB3VAR03, (**a3**) HB3VAR21, or (**a4**) IT4VAR13. (**a**) Statistical significance was determined using unpaired Mann–Whitney test to compare D0 and D30 samples, Black lines indicate the median and interquartile range. (**b**) Statistical significance was determined using Wilcoxon matched pairs signed rank test and significant *P*-values shown along the top of the panels. Black lines indicate the median and interquartile range. (**c**) The percentage phagocytosis of individual samples was correlated to the antibody reactivity (EU %) of each individual sample (day 0) using Spearman correlation test. Spearman´s correlation coefficient (r_s_) and *P-*value are shown in each panel. Red dots (a, c) and red lines (b) represent SM samples (CM and nCSM). (**d**) The percentage of phagocytosis shown for each of the two clinical categories uncomplicated malaria (UM) and severe malaria (CM and nCSM) at day 0 and day 30. Box blot with 5 to 95% percentile. The multiple comparisons were performed by using a mixed-effect model (REML) in GraphPad Prism 9. Statistical significance is shown along the top of the panels.

ADCP of merozoites has been suggested to contribute to protective immunity in humans, and the opsonic phagocytosis assay has been shown as a valuable technique to assess anti-malarial immunity^22^. We optimized a bead-based ADCP assay from the protocol described by Lloyd et al.^23^ using undifferentiated CD64^+^ (FcγRI), CD32^+^ (FcγRII) and CD16^-^ (FcγRIII) THP-1 cells (Fig. S1). Neutravidin and fluorescently labelled beads coupled with biotinylated DBLβ protein (PFD1235w, HB3VAR03, HB3VAR21, IT4VAR13) domains were pre-incubated with plasma from our Beninese donors (positive controls, i.e., a pool of highly reactive samples from Beninese individuals) or from negative control donors without exposure to *P. falciparum* parasites. THP-1 cells were added, and bead uptake by the cells was estimated by flow cytometry (Fig S2). The presence of malaria-specific IgG had no or little effect on the uptake of uncoupled BSA-beads nor did the presence of malaria naïve IgG affect the uptake of coupled beads by THP-1 cells (Fig. S2b). This demonstrates that the phagocytosis of beads by the THP-1 cells depended on opsonization by antigen-specific IgG. Having established the optimal dilution of plasma to be 1:200 (Fig. S2g, h), we measured the functional activity of antigen-specific plasma IgG antibodies. Except for HBVAR03 DBLβ (*P* = 0.68), the convalescent (day 30) Beninese plasma samples promoted a stronger phagocytosis of DBLβ-coated beads (PFD1235w and IT4VAR13 DBLβ, *P* < 0.0001) than samples obtained on the day of hospitalization (Fig. 5a1–a4). This was also seen for matched samples (Fig. 5b1–b4).

The anti-DBLβ IgG levels against PFD1235w and HB3VAR03 (group A), and against IT4VAR13 (group B), were positively correlated with the ADCP activity against the DBLβ domains (PFD1235w, *r* = 0.48, *P* < 0.001; HB3VAR03, *r* = 0.50, *P* < 0.001; IT4VAR13, *r* = 0.39, *P* < 0.001), suggesting that this type of PfEMP1-variants are major targets of IgG promoting ADCP (Fig. 5c1–c4). To further evaluate the significance of PfEMP1 as a target of acquired functional antibodies, and to examine whether responses to specific PfEMP1 domains might be important in protection from severe disease, we stratified the data based on the clinical category of the children. The level of IgG to each of the four DBLβ domains (PFD1235w, HB3VAR03, HB3VAR21, IT4VAR13) were similar in the UM and SM (Fig. 3), however in the functional assay, most samples from UM children showed a marked increase in phagocytosis activity against IT4VAR13 compared to those from SM children at day 0 (Fig. 5d4), but this did not differ for PFD1235w, HB3VAR03, and HB3VAR21 (Fig. 5d1–d3).

## Discussion

*Plasmodium falciparum* causes the most severe form of malaria and expresses PfEMP1 proteins on the surface of IEs. These proteins are expressed in a mutually exclusive manner and adhere to a range of vascular receptors that facilitate IE evasion of splenic clearance^24,25^. Severe malaria has been linked to IE adhesion via specific host receptors and is mediated by structurally related PfEMP1 (reviewed in^11,26^). In malaria endemic areas with stable transmission of *P. falciparum,* protective immunity is acquired during childhood, first to severe complications and later to clinical disease^27,28^. This is believed to be a result of an ordered acquisition of antibodies, with antibodies to a relatively conserved set of PfEMP1 proteins associated with severe disease acquired prior to antibodies to a larger and more diverse set of PfEMP1 proteins associated with uncomplicated malaria and asymptomatic parasitemia^27,29^.

In Beninese children, we found that IgG reactivity against ICAM-1-binding DBLβ did not differ among children of similar age with severe or uncomplicated malaria (Fig. 2), and that IgG levels were reduced 30 days following hospitalization (Fig. 1). In agreement with this, our recent study found that Ghanaian children show a transient increase in IgG reactivity to ICAM-1-binding DBLβ domains two weeks after acute malaria followed by decreased levels six weeks later. As in the present study, our previous work^30^ and that of others^16,31^ did not observe differences in IgG reactivities to DBLβ domains between children of similar age with severe or uncomplicated malaria.

The clinical significance of PfEMP1-specific antibodies is thought to involve their ability to interfere with sequestration of IEs in various tissues including DBLβ-specific-IgG that inhibit IE adhesion to ICAM-1^9,14,32,33^. Here, we investigated the activity of naturally acquired functional antibodies from children who were exposed to *P. falciparum* infection, i.e., we looked at adhesion-inhibitory antibodies and antibodies mediating ADCP. Our findings show that antibodies from participants included in the study were able to not only react with the domains, but that the IgG antibodies have functional activity as they elicited inhibitory effects ranging from 16 to 78% upon the ICAM-1-binding of DBLβ domains encoded by dual-receptor binding PfEMP1 (Fig. 4), and promoted opsonic phagocytosis (Fig. 5). These findings support previous work, which shows that the PfEMP1 family is an important target of protective antibodies against malaria^5,10,34–37^. When stratifying our study participants into distinct clinical categories to assess the potential inhibitory role of their antibodies, plasma from children with UM showed higher inhibition of PFD1235w DBLβ binding to ICAM-1 compared to plasma from SM children, including CM cases (Fig. 4). Thus, anti-PFD1235w IgG antibodies can inhibit PfEMP1 binding and might prevent PFD1235w-expressing IEs (or close variants thereof) from binding to ICAM-1 on endothelial cells, thus providing protection against developing SM in these children. if that hypothesis can be confirmed in future studies, it would indicate that inclusion of peptides based on this domain in malaria vaccine cocktails would be warranted. Cytophilic antibodies, IgG1 and IgG3 are known for their high affinity for most of the Fc receptors on diverse immune cells and their function in opsonization for effector cell function^38–41^. As in other studies^42,43^, the IgG antibody response was dominated by IgG1 and IgG3 (Fig. 3), which facilitate protection against malaria through cell-mediated mechanisms, such as ADCP and antibody-dependent cellular inhibition. In contrast, IgG2 and IgG4 have been classically considered as non-protective antibodies against malaria because they poorly engage Fc receptors^41^, and levels of PfEMP1-specific IgG of these sub-classes IgG2 and IgG4 isotype levels were low in our study participants (data not shown). Besides neutralization of pathogens by antibodies, phagocytosis is considered as one of the most important anti-pathogen activities^44^. ADCP involves receptor-mediated interactions between immune effector cells and the Fc domain of cytophilic IgG bound to pathogen^45^. Here, we measured opsonic phagocytosis activity using undifferentiated THP-1 monocytes and found that children with UM and SM had similar levels of IgG to the different DBLβ domains, but plasma from UM children (day 0) showed significantly higher percentage phagocytosis activity to group B IT4VAR13 DBLβ (Fig. 5d4). This could indicate that a higher opsonic phagocytosis activity of plasma against DBLβ domain is associated with protection from developing SM, a finding that will be of interest to investigate further in future studies.

## Methods

### Plasma and parasite samples

The study protocol was reviewed and approved by the Comité National d’Ethique pour la Recherche en Santé (CNERS), No. 87/MS/DC/SGM/DRFMT/CNERS/SA Cotonou, République du Benin. All methods used in this study were performed in accordance with relevant guidelines and regulations.

Plasma samples (*n* = 137) were collected at three hospital centers in the cities of Cotonou, Southern Benin during the malaria transmission season of June–September 2019. After informed consent had been obtained from a parent or a legal guardian, children less than 5 years of age were subjected to clinical investigation and screened by malaria rapid diagnostic test (Malaria Pf/Pan, DiaQuick). Venous blood samples were collected from the enrolled children and parasitemia quantified by microscopy. Blood samples were collected from each child on the day of hospitalization (disease presentation) and 30 days following discharge (convalescent sample). Clinical manifestations were classified according to the definitions by the World Health Organization^46^. Patients were categorized as having cerebral malaria (CM; *n* = 34) if they had a positive blood smear of *P. falciparum* and unarousable coma (Blantyre coma score [BCS] ≤ 2) with exclusion of other causes of coma. Patients were categorized as having non-cerebral severe malaria (nCSM; *n* = 51) if they presented with hyperparasitemia (> 50,000), and/or severe anemia (hemoglobin < 5 g/dL), and no coma. Patients with uncomplicated malaria (UM; *n* = 52) had fewer parasites than 50,000 parasites per µL accompanied by fever, headache, or myalgia without signs of severity and evidence of vital organ dysfunction.

A pool of plasma from *P. falciparum*-exposed Liberian adults^47^, or hyperimmune Beninese children, and 30 non-exposed Danish individuals were used in ELISA as positive and negative controls, respectively.

The *P. falciparum* parasite clones PFD1235w, HB3VAR03, IT4VAR13 and HB3VAR21 (KOB63129) were maintained in long-term in vitro culture and selected for IE surface expression by repeated antibody selection as described^48–50^. The identity of isolates was routinely verified by genotyping as described^51^, and *Mycoplasma* infection was excluded by using the MycoAlert *Mycoplasma* detection kit (Lonza) according to the manufacturer´s instruction.

### Recombinant proteins

Seven ICAM-1-binding group A (PFD1235w, PF11_0521, Dd2VAR32, KJ866957, KJ866958, AFJ66668, KM364031, HB3VAR03) and four group B ICAM-1-binding DBLβ protein domains (IT4VAR13, HB3VAR21/KOB63129, PFL0020w, Dd2VAR01A), and three group A non-ICAM-binding DBLβ protein domains (Dd2VAR25, Dd2VAR52, HB3VVAR01) were expressed as His-tagged proteins in *Escherichia coli* Shuffle C3030 cells (New England BioLabs). The protein domains (Table S1) were purified by immobilized metal ion affinity chromatography using HisTrap HP 1-ml columns (GE Healthcare) as described previously^27,30,32^. Recombinant Fc-tagged ICAM-1 was expressed in HEK293 cells and purified as described previously^52^.

### *P. falciparum* DBLβ-reactive antibodies

Maxisorp microtiter plates (Sigma-Aldrich) were coated with recombinant DBLβ domains (50 µL; 2 µg/mL) in 0.1 M glycine/HCl buffer pH 2.75 overnight at 4ºC as described previously^32^. Following a washing step in wash buffer (PBS, 0.5 M NaCl, 1% Triton X-100, pH 7.4) plates were blocked using blocking buffer (PBS, 0.5 M NaCl, 1% Triton-X-100, 1% BSA, 0.03 mM phenol red, pH 7.2). Plasma samples (diluted 1:200 in blocking buffer) were incubated (50 µL/well, 1 h, room temperature) in duplicate wells. The plates were washed and bound IgG antibody was detected with horseradish peroxidase (HRP)-conjugated anti-human IgG (1:3,000 in blocking buffer) (Agilent). Following incubation (1 h) and washing as described above, bound detection antibody was visualized using TMB according to the manufacturer´s instructions (Agilent). The reaction was stopped by adding 0.2 M H_2_SO_4_ (50 µL/well) and the optical density (OD) values were read at 450 nm using a VERSAmax microplate reader (Molecular Devices). Antibody reactivity was expressed as ELISA units calculated as (OD_sample_ − OD_background_)/(OD_positive control sample_ – OD_background_) × 100%^53^.

### DBLβ-specific IgG Subclass levels

The anti-DBLβ IgG subclass reactivity was measured in plasma samples collected from children with malaria. Maxisorp plates (Nunc, Thermofisher Scientific, Denmark) were coated with different recombinant DBLβ domains (50 µL/well; 2 µg/mL; 0.1 M glycine/HCl buffer pH 2.75) overnight at 4 °C. The plates were emptied and washed once with washing buffer and blocked with blocking buffer as described above. Plasma samples (50 µL; 1:50 in blocking buffer) were added duplicate wells. Following incubation (1 h) and washing as described above, bound antibody was detected with mouse HRP-conjugated anti-human IgG (anti-Human IgG1, IgG2, IgG3, or IgG4; Sigma-Aldrich) (1:2000 dilution in blocking buffer; 50 μL/well). Following 1 h of incubation and subsequent three times washing, plates were developed, colour reactions stopped, OD values read, and results calculated as described above.

### Antibody-mediated inhibition of DBLβ binding to ICAM-1

Inhibition of recombinant DBLβ domain binding to ICAM-1 by participants’ plasma was measured by ELISA. Briefly, wells of Maxisorp plates were coated with recombinant Fc-tagged ICAM-1 (50 µL; 2 μg/mL; 0.1 M glycine/HCl buffer pH 2.75) by incubation overnight at 4ºC and blocked with blocking buffer (1 h; room temp). His-tagged recombinant DBLβ domains (0.5–16 µg/mL) were mixed with immune plasma (1:10 final concentration) and subsequently added to duplicate wells (1 h; room temp) of ICAM-1 coated plates. Following washing, bound DBLβ protein was detected using HRP-conjugated anti-penta-His antibody (1:3,000) (Qiagen). Following 1 h incubation and three rounds of washing, bound antibody was visualized using TMB and plates read as described above.

### Phagocytosis assay

The DBLβ domains were biotinylated and coupled to Neutravidin-labelled fluorescent beads (1.0 µm microspheres; yellow-green fluorescent 505/515, 1% solids) in a ratio of 10^7^ beads per mg DBLβ protein. Briefly, beads were resuspended by vortexing in 400 µL of ddH2O, biotinylated DBLβ protein (1 mg/ml) was added to the beads, mixed by vortexing and incubated at room temperature (4 h; 100 rpm). Coupled beads were pelleted at 18,000 × g, washed twice in 1 mL of dilution buffer (PBS containing 1% BSA, sterile), and resuspended in 1000 µL dilution buffer and stored in the dark at 4ºC until used. On the day of assay, U-bottom plates were blocked (0.1% BSA in PBS, 150 µL/well) at room temperature for at least 30 min. For opsonization, this was followed by incubation of heat-inactivated plasma (40 µL of 1:200 dilution) with 10^5^ DBLβ-coupled beads (10 µL). To prevent photobleaching, plates were covered with foil and placed on a shaker for 1 h at 100 rpm. Following two washes with 1 × PBS, beads were incubated 45 min with 5 × 10^4^ THP-1 cells (beads-to-monocyte ratio of 2:1, viability of THP-1 cells > 95% at the beginning of the assay) in 50 µL of RPMI-1640 at 37ºC and 5% v/v CO_2_. To stop phagocytosis, 200 µL of chilled PBS was added to each well of the plates and immediately placed on ice or at 4 °C. Samples were immediately read on FACS BD Accuri C6-plus (BD Biosciences), and THP-1 cells were gated by size, and granularity on FSC and SSC plots and fluorescence in the FITC-channel. A minimum of 5,000 THP-1 cells were recorded for each sample. Data were analyzed using the FlowJo (ver 10.6.0) software. Percentage phagocytosis was the proportion of THP-1 cells that were positive for fluorescence in the FL-1 channel. THP-1 cells in two major fluorescence peaks were visualized by setting the FL-1 channel against the FL-4 channel.

### Statistical analysis

Wilcoxon rank sum test was used to compare antibody levels in matched and paired data groups between the time of hospitalization and at convalescence. We used Kruskal-Walli’s test with Dunn´s multiple comparison test of functional data for children in different clinical categories i.e., CM, nCSM, and UM. Differences were considered significant when *P* < 0.05. GraphPad Prism 9.0 was used for the analysis.

## Supplementary Information

Supplementary Information.

## Data Availability

The datasets used and analyzed during the current study are available from the corresponding author on request.
