# Supplementary material for: ICAM-1-binding Plasmodium falciparum erythrocyte membrane protein 1 variants elicits opsonic-phagocytosis IgG responses in Beninese children
Source: Sci Rep. 2022 Jul 29;12:12994. doi: 10.1038/s41598-022-16305-0 (PMC9338288; doi:10.1038/s41598-022-16305-0)
Supplement: Supplementary file 1 — Supplementary Information. [file 41598_2022_16305_MOESM1_ESM.docx]

**Supplementary information**

Supplementary Figure S1


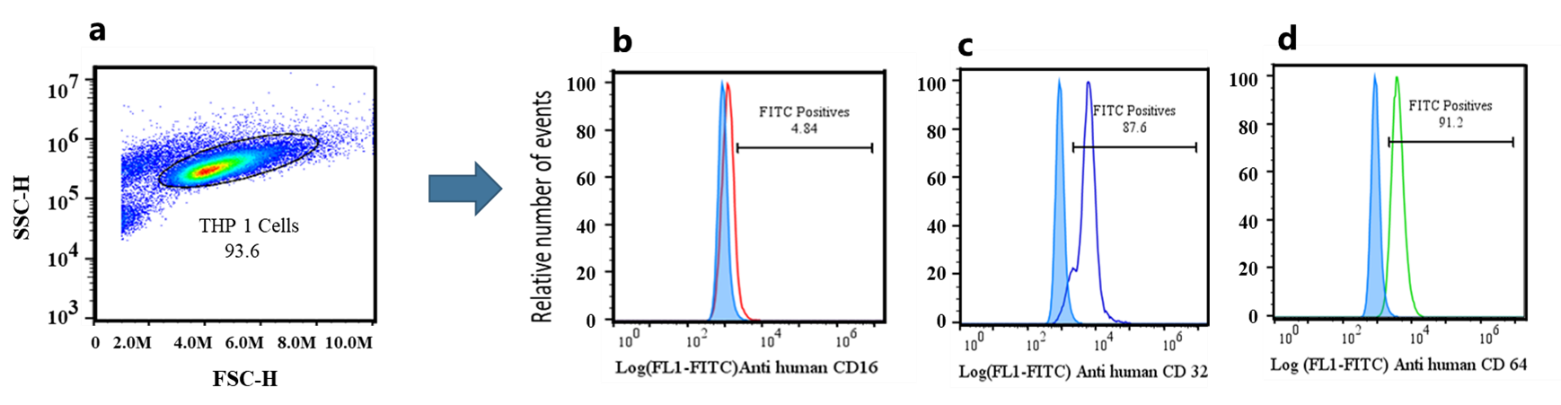


**Figure S1.** **Fcγ receptors on the THP-1 cell surface.** The assay was optimized using undifferentiated THP-1 cells, that only express CD64 (FcγR I), CD32 (FcγR II), and not CD16 (FcγR III). THP-1 cells were stained with; (**a**) THP-1 cells were gated on light scattered characteristics;(**b**) FITC anti-CD16 (red), (**c**) anti-CD32 (blue) and (**d**) anti-CD64 (green) antibodies and visualized by flow cytometry. Unstained cells are shown in shaded blue. THP-1 monocytes were maintained below 5×10^5^ cells per ml of culture to ensure consistent surface receptor expression. Prior to use, THP-1 cells were immuno-stained for the above receptors to confirm their surface phenotype. The viability of THP-1 cells was >95 % at the beginning of the assay.

Supplementary Figure S2


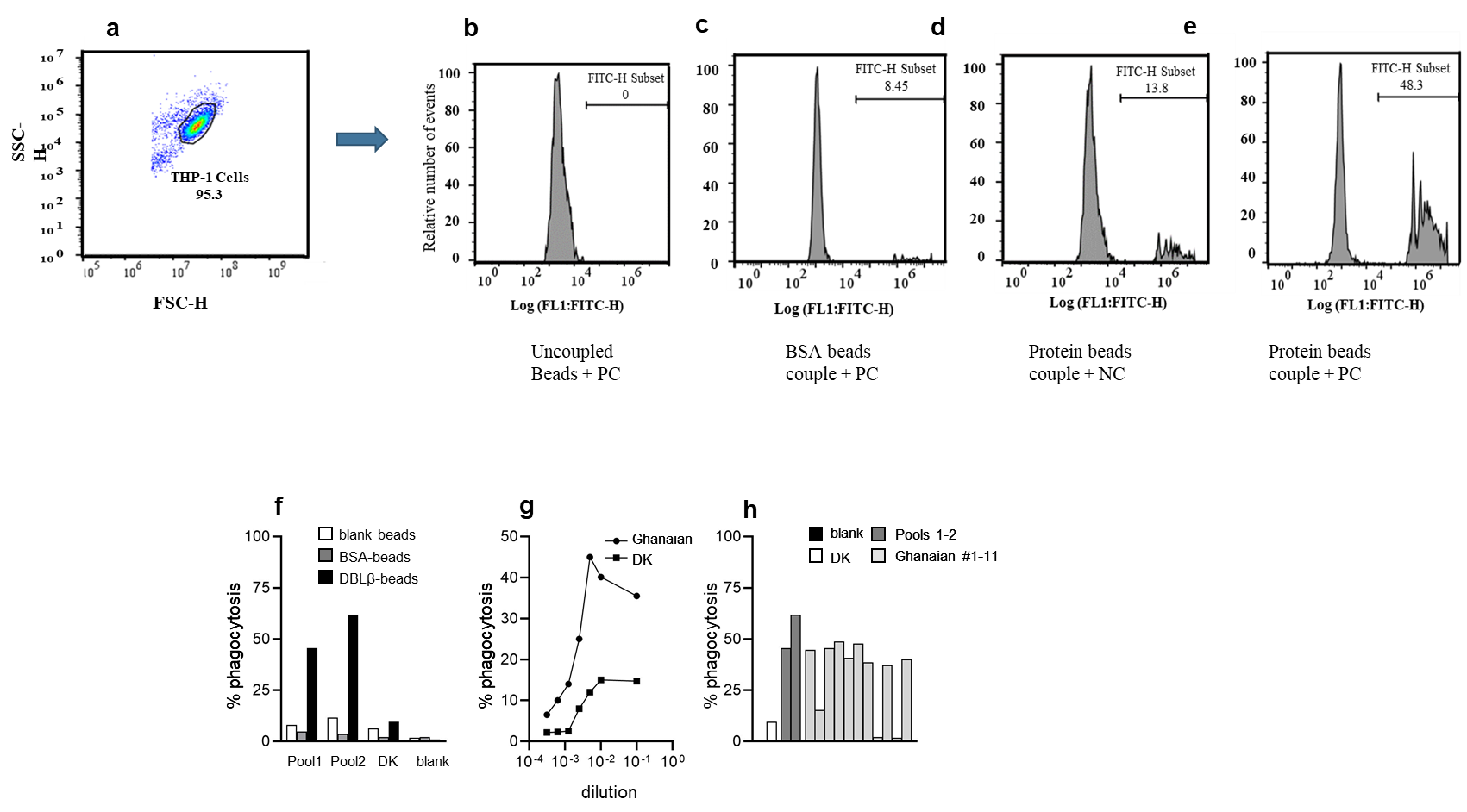


**Figure S2.** **Phagocytosis of PfEMP1 domains after opsonization with anti-PfEMP1 antibodies from plasma.** Biotinylated DBLβ domains were immobilized on the surface of Neutravidin labelled fluorescent beads and incubated with plasma. Opsonized beads were added to THP-1 cells and the bead uptake was estimated by flow cytometry. (**a)** THP-1 cells were gated on light scattered characteristics (**b**) uncoupled beads incubated with positive control. (**c**) BSA coupled beads incubated with positive control. (**d**) Biotinylated DBLβ domain-coupled beads incubated with plasma from Danish blood donors (nc, negative control) never exposed to *P. falciparum* and **(e**) Biotinylated DBLβ domain-coupled beads incubated with plasma from malaria exposed children (pc, positive control). Plasma from these children promoted strong phagocytosis of DBLβ-coated beads while there was little effect of malaria-specific antibodies on the uptake of either uncoupled or BSA- beads, demonstrating that phagocytosis is antigen-specific. More so there was a negligible uptake of coupled beads by malaria naïve donors demonstrating that phagocytosis is malaria specific**.** (**f**). Biotinylated DBLβ domain-coupled beads were incubated with either pooled plasma from children with uncomplicated malaria with high level of IgG to group A PFD1235w; HB3VAR03, and group B IT4VAR13; HB3VAR21 DBLβ domains or plasma from malaria naïve individuals (DK) as negative control. (**g**) Phagocytosis (%) depend on the titer of plasma dilution. Pooled Ghanaian and Danish plasma were diluted 1:10 to 1:3,200 using 0.1% BSA in PBS. The percentage of THP-1 cells that internalized Biotinylated DBLβ domain-coupled beads declined with increasing plasma dilution, confirming a relationship between amount of IgG to the domain in plasma and phagocytic activity. Plasma dilutions that gave the best discrimination among the samples were 1:200. (**h**). Phagocytosis (%) mediated by plasma (1:200 dilution) from 11 Ghanaian children, a Danish plasma (DK pool), and a pool of plasma from Ghanaian children (same as in b). The assay conditions enabled resolution of differences between individuals, detecting low, high or intermediate responses.

Supplementary Table S1: Recombinant proteins in the study

**Genome PfEMP1 Domain subtype Binds ICAM-1 Group References**

MN35 KJ866957 (MN035) DBLβ3_D4 Yes A ^1,2^

Dd2 Dd2VAR32 DBLβ3_D4 Yes A ^1-5^

3D7 PF11_0521 DBLβ3_D4 Yes A ^1,2,4,6^

3D7 **^a^**PFD1235w DBLβ3_D4 Yes A ^2,7-9^

MN56 KM364031 DBLβ1_D4 Yes A ^9^

AA39 KJ866958 DBLβ3 Yes A ^2,9^

1914 AFJ66668 DBLβ1_D4 Yes A ^2,9^

IT4 **^b^**IT4VAR13 DBLβ3_D4 Yes B ^2,4,9-11^

3D7 PFL0020w DBLβ5_D4 Yes B ^4,11^

Dd2 **^c^**Dd2var01A DBLβ5_D4 Yes B ^4,11^

HB3 **^d^**HB3VAR21 DBLβ5_D4 Yes B ^11^

Dd2 Dd2VAR25 DBLβ11_D4 No A ^4^

Dd2 Dd2VAR52 DBLβ7_D4 No A ^4^

HB3 HB3VAR01 DBLβ7_D4 No A ^4^

HB3 HB3VAR03 DBLβ Yes A ^2,7,9^

**^a^** Also known as PF3D7_0425800

**^b^** Also known as ABM88750

**^c^** Also known as AAA75396

**^d^** Also known as KOB63129.1

References

1 Lennartz, F. *et al.* Structure-Guided Identification of a Family of Dual Receptor-Binding PfEMP1 that Is Associated with Cerebral Malaria. *Cell Host Microbe* **21**, 403-414, doi:10.1016/j.chom.2017.02.009 (2017).

2 Lennartz, F., Smith, C., Craig, A. G. & Higgins, M. K. Structural insights into diverse modes of ICAM-1 binding by <em>Plasmodium falciparum</em>-infected erythrocytes. *Proceedings of the National Academy of Sciences* **116**, 20124-20134, doi:10.1073/pnas.1911900116 (2019).

3 Jensen, A. T. *et al.* Plasmodium falciparum associated with severe childhood malaria preferentially expresses PfEMP1 encoded by group A var genes. *The Journal of experimental medicine* **199**, 1179-1190 (2004).

4 Rask, T. S., Hansen, D. A., Theander, T. G., Gorm Pedersen, A. & Lavstsen, T. Plasmodium falciparum erythrocyte membrane protein 1 diversity in seven genomes–divide and conquer. *PLoS computational biology* **6**, e1000933 (2010).

5 Lau, Clinton K. Y. *et al.* Structural Conservation Despite Huge Sequence Diversity Allows EPCR Binding by the PfEMP1 Family Implicated in Severe Childhood Malaria. *Cell Host & Microbe* **17**, 118-129, doi:<https://doi.org/10.1016/j.chom.2014.11.007> (2015).

6 Oleinikov, A. V. *et al.* High throughput functional assays of the variant antigen PfEMP1 reveal a single domain in the 3D7 Plasmodium falciparum genome that binds ICAM1 with high affinity and is targeted by naturally acquired neutralizing antibodies. *PLoS pathogens* **5**, e1000386 (2009).

7 Joergensen, L. *et al.* Surface co-expression of two different PfEMP1 antigens on single Plasmodium falciparum-infected erythrocytes facilitates binding to ICAM1 and PECAM1. *PLoS pathogens* **6**, e1001083 (2010).

8 Bengtsson, A. *et al.* A novel domain cassette identifies Plasmodium falciparum PfEMP1 proteins binding ICAM-1 and is a target of cross-reactive, adhesion-inhibitory antibodies. *The Journal of Immunology* **190**, 240-249 (2013).

9 Lennartz, F. *et al.* Structure-guided identification of a family of dual receptor-binding PfEMP1 that is associated with cerebral malaria. *Cell host & microbe* **21**, 403-414 (2017).

10 Janes, J. H. *et al.* Investigating the host binding signature on the Plasmodium falciparum PfEMP1 protein family. *PLoS pathogens* **7**, e1002032 (2011).

11 Olsen, R. W. *et al.* Acquisition of IgG to ICAM-1-binding DBLβ domains in the Plasmodium falciparum erythrocyte membrane protein 1 antigen family varies between groups A, B, and C. *Infection and immunity* **87**, e00224-00219 (2019).
